# Victoria Hospital for Children, Queen's Road, Chelsea

**Published:** 1902-05-31

**Authors:** 


### VICTORIA HOSPITAL FOR CHILDREN, QUEEN'S ROAD, CHELSEA.

Little over a quarter of an hour sufficed for the despatch of business at the annual meeting of the governors of this hospital, which took place on May 21st.

The Chairman (Mr. Martin R. Smith) was unfortunately unable to be present on account of illness, but a letter from him was read to the meeting by the Secretary (Mr. H. G. Evered). After expressing regret at his inability to attend the meeting, Mr. Smith in his letter went on to say that their kind patroness, H.R.H. Princess Louise, had graciously promised to preside at the open air fête which was to be held on behalf of the hospital on July 2nd. They were also deeply indebted to General Sir Henry Norman for kindly permitting the fête to be held in the gardens of the Royal Chelsea Hospital. It was with feelings of great thankfulnesss that the writer was able to inform the meeting that the amount collected for the construction of new wards, or rather of a new hospital, was sufficient to justify their commencing work forthwith. If their work had been useful in the past how much more would it be in the future. All chairmen of all hospitals considered theirs the most deserving of support, but at any rate the Victoria Hospital appealed to all that was greatest and tenderest in human nature. There was no district in London where rich and poor were more curiously mixed than in South Kensington and Chelsea. It was satisfactory to note that there had been a slight increase in the annual subscriptions, amounting to £162, this undoubtedly representing 100 new subscribers. The chief event of the past year had been their festival dinner, presided over by Field-Marshal Earl Roberts, when the fund for rebuilding had advanced from £6,000 to £12,000. The writer felt that it was incumbent upon them to tender their earnest and grateful thanks to Lord Roberts. It was a matter for regret that there was a deficiency of £650 in the finances of the home at Broadstairs, which would have to be made good by the hospital at Chelsea. The Broadstairs home was of the greatest possible service in completing the work begun in London. The hospital did not for a moment grudge the cost of the home, which was most admirably conducted by the matron, Mrs. Day; it was, however, most earnestly to be desired that all subscribers would add a few shillings to their subscriptions for the benefit of the home. The election of life Governors then took place, and the retiring members of the Committee of Management having been re-elected, the proceedings came to a close.

